# MicroRNAs as Potential Biomarkers in Atherosclerosis

**DOI:** 10.3390/ijms20225547

**Published:** 2019-11-07

**Authors:** Alexey Churov, Volha Summerhill, Andrey Grechko, Varvara Orekhova, Alexander Orekhov

**Affiliations:** 1 Institute of Biology of the Karelian Research Centre, Russian Academy of Sciences, 11 Pushkinskaya Street, Petrozavodsk Karelia 185910, Russia; achurou@yandex.ru; 2 Institute for Atherosclerosis Research, Skolkovo Innovative Center, Moscow 121609, Russia; 3 Federal Research and Clinical Center of Intensive Care Medicine and Rehabilitology, 14-3 Solyanka Street, Moscow 109240, Russia; avg-2007@yandex.ru; 4 Laboratory of Angiopathology, Institute of General Pathology and Pathophysiology, 8 Baltiyskaya Street, Moscow 125315, Russia; varvaraao@gmail.com; 5 Institute of Human Morphology, 3 Tsyurupa Street, Moscow 117418, Russia

**Keywords:** atherosclerosis, miRNA, biomarker, inflammation, endothelial dysfunction, endothelial cells, vascular smooth muscle cells, lipid metabolism

## Abstract

Atherosclerosis is a complex multifactorial disease that, despite advances in lifestyle management and drug therapy, remains to be the major cause of high morbidity and mortality rates from cardiovascular diseases (CVDs) in industrialized countries. Therefore, there is a great need in reliable diagnostic/prognostic biomarkers and effective treatment alternatives to reduce its burden. It was established that microRNAs (miRNAs/miRs), a class of non-coding single-stranded RNA molecules, can regulate the expression of genes at the post-transcriptional level and, accordingly, coordinate the cellular protein expression. Thus, they are involved not only in cell-specific physiological functions but also in the cellular and molecular mechanisms of human pathologies, including atherosclerosis. MiRNAs may be significant in the dysregulation that affects endothelial integrity, the function of vascular smooth muscle and inflammatory cells, and cellular cholesterol homeostasis that drives the initiation and growth of an atherosclerotic plaque. Besides, distinct expression patterns of several miRNAs are attributed to atherosclerotic and cardiovascular patients. In this article, the evidence indicating the multiple critical roles of miRNAs and their relevant molecular mechanisms related to atherosclerosis development and progression was reviewed. Moreover, the effects of miRNAs on atherosclerosis enabled to exploit them as novel diagnostic biomarkers and therapeutic targets that may lead to better management of atherosclerosis and CVDs.

## 1. Introduction

Atherosclerosis is the common cause of ischemic heart disease, stroke, and sudden death. These conditions are responsible for the total mortality of over 50%, in technologically advanced countries [1]. It is a complex multifactorial disease characterized by the formation of lesion associated with the subendothelial lipid accumulation and the low-grade inflammation in the walls of large- and medium-sized arteries. Subendothelial lipid accumulation, in particular, accumulation of modified low-density lipoprotein (LDL) particles is the main inducing process of atherogenesis, therefore, it is a crucial event in the development of atherosclerotic lesions [2]. The sub-endothelial lipid accumulation is enabled by the loss of endothelial integrity, the key regulator of the vascular system homoeostasis, which compromises vasodilation, and plays both pro-inflammatory and prothrombotic roles; and therefore determines the progression of early atheroma. Retained lipoproteins cause local biological reactions, including a chronic and maladaptive macrophage- and T-cell-governed inflammatory responses that promote subsequent lesion development, i.e., atherosclerotic plaque formation, progression, and rupture [3]. In particular, monocyte-derived macrophages uptake modified LDL and develop into so-called foam cells engaging further T- and B- inflammatory cells into the expanding layer of the arterial intima. In fact, inflammatory mechanisms mediate all stages of the atherosclerotic lesion development by coupling up dyslipidemia to the formation of complex vulnerable plaques that are responsible for the clinical complications of atherosclerosis, namely, acute myocardial infarction (AMI) or stroke [4]. Moreover, both the innate and adaptive immune systems become intimately involved in the development of atherosclerotic plaque [5]. It is worth noting that atherosclerotic lesions are generally spared at the straight parts of the arterial tree but are found predominantly at branch points, where blood flow is disturbed by a limited forwarding direction [6].

Developing in a multi-step fashion, atherosclerosis is a disease of slow progress (over several decades) and it generally remains unnoticed prior to the manifestation of its first clinical symptoms. Therefore, in many cases, the first clinical manifestations of atherosclerosis appear when the lesion is already well developed causing significant narrowing of the vascular lumen and that may lead to fatal consequences [7]. Despite it being considered to be prevalent in older people, numerous studies reported the presence of silent atherosclerotic lesions in a wide population of young adults, moreover, the mortality rate of subclinical atherosclerosis can reach up to 100% in this cohort of subjects [8,9,10,11,12,13,14]. The presence of pro-atherogenic changes in the blood was also reported in children [15]. Thus, the atherosclerotic process begins in childhood, with the silent progress through a long preclinical phase, and ultimately manifests clinically, typically from middle age. Therefore, it is important to find new approaches for better understanding of atherosclerosis pathogenesis and to establish reliable diagnostic biomarkers and therapeutic strategies for each stage of the atherosclerotic process. In that regard, regulatory microRNAs (miRNAs/miRs)—as the essential part regulating various pathomechanisms of atherosclerotic plaque development—have attracted special attention. In this review, we will discuss the evidence indicating that cellular and molecular processes associated with atherosclerosis pathophysiology are affected by numerous miRNAs that, accordingly, can be used as diagnostic biomarkers and therapeutic targets for the development of novel therapies.

## 2. Biological Role of miRNAs

MiRNAs are an abundant class of highly conserved single-stranded non-coding endogenous RNAs of ~22 nucleotides (nt) in length, which negatively regulate expression of genes at the post-transcriptional level by inhibiting translation of protein from the messenger (mRNA) or promoting its degradation [16,17]. They function by the direct base-pairing to the 3′ untranslated region (3′ UTR) of specific target mRNA sequences. Consequently, they play an important role in forming the transcriptomes and proteomes of eukaryotic organisms. It was estimated that the human genome encodes at least 800 miRNAs [18], although, the total number of miRNAs is unknown. Most of the miRNA genes located in the introns of protein-coding genes [19] controlling 30% of protein-coding genes [20]. The important role of miRNAs, as vigorous regulators of many biological processes, including cell growth, proliferation, differentiation, migration, senescence, apoptosis, and angiogenesis, is widely recognized [21,22]. Moreover, aberrant expression and dysregulation of miRNA function are closely linked to the following human pathologies: cancer, diabetes, obesity, atherosclerosis, and CVDs [23,24,25,26,27].

### MiRNA Biogenesis

**Figure 1:**
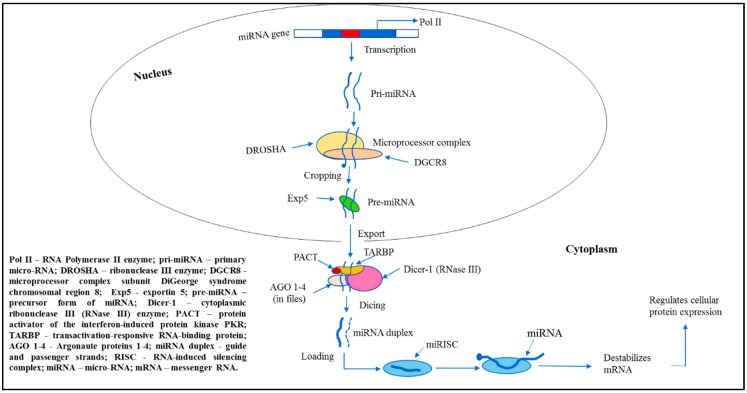
MiRNA biogenesis: Canonical pathway.

Mechanisms of miRNA biogenesis and function were established only recently (over the past decade) [28]. The canonical miRNAs biogenesis pathway is characterized by microprocessor complex subunit DiGeorge syndrome chromosomal region 8 (*DGCR8)*, ribonuclease III (RNase III) enzyme DROSHA and Dicer processing (Figure 1).

Thus, in the nucleus of mammalian cells, most of miRNAs genes are transcribed by RNA Polymerase II (RNA Pol II) producing the long primary miRNAs (pri-miRNAs), 500–3000 base pair molecules [29]. The miRNA transcription depends on the host gene [30]. The subsequent folding of pri-miRNA specific regions into hairpin structures is a key aspect of the initial pri-miRNA processing. In turn, long pri-miRNA transcripts are cleaved by a microprocessor multiprotein complex containing RNA-binding proteins—such as DGCR8 and DROSHA—thus, generating pre-miRNAs, the miRNA precursor form [31]. Pre-miRNAs are hairpin structures that are formed by this cleavage. Then, pre-miRNAs are transported to the cytoplasm by exportin 5 (Exp5) protein [32,33,34], where cytoplasmic RNase III Dicer-1 subsequently cleaves them into unstable mature asymmetric duplex miRNAs of ∼22-nt in length with the hairpin removed [35]. One strand of the duplex, usually with relatively lower stability of base-pairing at the 5-end (“the thermodynamic asymmetry rule”), is intended to become the mature guide miRNA [36]. Its phosphorylated 5-end is essential for the interaction with an Argonaute (AGO) proteins [37]. The other strand was referred as passenger strand. Both strands undergo further processing mediated by specific proteins. Thus, in the cytoplasm, selectively associated with AGO proteins, the guide strand of miRNA duplex is getting incorporated into the RNA-induced silencing complex (RISC) that is facilitated by Dicer-1/transactivation-responsive RNA-binding (TRBP) protein, as well as PACT protein, the activator of interferon-induced protein kinase R (PKR) [38]. The passenger strand is either degraded by linked to RISC AGO proteins or performs important regulatory functions for the guide strand. Several authors presented reports on the functional activity of passenger strands [39,40]. The RISC integrated active single-stranded miRNA represses mRNA translation, i.e., destabilizes mRNA transcripts by cleavage or deadenylation, thus, regulates protein expression. 

However, there are deviations in this canonical pathway of miRNA biogenesis: some subclasses of miRNAs are derived by alternative biogenesis pathways, thereby, supporting an additional level of complexity to miRNA-dependent regulation of gene expression. The non-canonical miRNA biogenesis pathways were described demonstrating the ability and flexibility of cells to produce pre-miRNA-like hairpins that are moved to Dicer and/or 5-phosphorylated small RNAs that bind straight to AGO proteins [41]. 

Overall, despite the limited knowledge about the upstream mechanisms controlling miRNA abundance, numerous studies confirmed that miRNA expression is controlled by Dicer and DROSHA processing complex [42,43,44]. Remarkably, the expression profiling studies indicated that the expression of many miRNAs is a tissue-specific or developmental stage-specific, and that is suggestive of a significant role of some miRNAs in tissue specification and cell lineage determination [45,46]. For example, during the development of thyroid carcinoma, regulating human telomerase reverse transcriptase miR-138 is spatially restricted to distinctive cell types, while its precursor is universally expressed in different tissues [47,48]. The diverse expression profiles in different cells suggest that miRNAs physiological functions could be different in different cells and tissues.

## 3. Roles of Various miRNAs in Developmental Pathomechanisms of Atherosclerosis

### 3.1. miRNAs in the Regulation of Endothelial Cell Function

It is well known that the exposure of the endothelium to various stimuli, such as hypoxia, proinflammatory cytokines, oxidative stress, hypertension, hyperglycemia, shear stress, ageing, or injury, compromises the function of endothelial cells resulting in their increased proliferation, migration, apoptosis, senescence, angiogenesis, and inflammation. Increasing evidence demonstrated that specific classes of miRNAs participate in the control of pathways regulating endothelial function, including maintenance of vascular integrity, proliferation, and migration of endothelial cells [49,50]. The study reported that miR-221 can be dysregulated in endothelial progenitor cells (EPCs) and involved in the regulation of their function [51]. It was found that EPC expression levels of miR-221 were significantly higher in subjects with atherosclerosis-related coronary artery disease (CAD), compared to healthy controls, and, moreover, the upregulated expression of this miRNA diminished the proliferative ability of EPCs [51]. EPCs support the endothelial function with new healthy endothelial cells replacing damaged or apoptotic cells [52,53]. Further studies on cell-specific effects of miR-221 and miR-222 revealed that these miRNAs can modulate angiogenesis of endothelial cells by targeting the stem cell factor receptor c-kit [54] and also, indirectly regulate the expression of endothelial nitric oxide synthase (eNOS) in vitro [55]. Of note, eNOS is predominantly responsible for nitric oxide generation in vascular endothelium [56]. Nitric oxide is a key regulator of growth and migration of endothelial cells, vascular remodeling, and angiogenesis; its impaired bioavailability is a hallmark of patients with atherosclerosis [57,58,59]. In advanced plaques, endothelial cell-driven angiogenesis leads to neo-vessel formation invading the intima, a process that is interrelated to plaque growth, destabilization, and rupture. Other miRNAs can regulate angiogenesis development. It was demonstrated that the endothelial cell-restricted miR-126, by reducing the expression of sprout-related protein 1 (Spred-1), can promote developmental angiogenesis in vivo, therefore, is deeply involved in the aid of endothelial dysfunction [60]. Whereas, the overexpression of miR-92a can block angiogenesis and reduce migration of endothelial cells in vitro and in vivo [61]. Also, by targeting vascular endothelial growth factor receptor 2 (VEGFR2) and fibroblast growth factor receptor 1 (FGFR1), miR-129-1 and miR-133 were able to suppress key factors of angiogenesis—such as proliferation rate, cell viability, and migration activity of human umbilical vein endothelial cells (HUVECs)—in vitro [62]. The functional role of miRNAs (miR-146a, miR-147, miR-126, and miR-155 among others) in vascular remodeling response to the development of plaque, an essential component of atherosclerotic disease, was also reviewed [63].

Furthermore, the regulation of specific miRNAs by shear stress can promote either vasculo-protective or pro-atherogenic effects in endothelial cells. According to the current understanding, laminar shear stress imposed directly on the endothelium stimulates considerable changes in gene expression by regulating the expression of various miRNAs. Induced by high shear stress mediating the atheroprotective function, several miRNAs, namely, miR-10a, miR-19a, miR-23b, miR-101, and miR-143/145 were identified [64]. For example, the downregulation of miR-92a by shear stress increased eNOS expression, whereas, the upregulation of miR-19a contributed to the shear stress-induced cellular proliferation inhibition [65]. Moreover, the low shear stress-induced expression of miR-21 lead to pro-inflammatory phenotype of endothelial cells [66]. Although, in HUVECs, in response to prolonged unidirectional shear stress, upregulated miR-21 displayed atheroprotective function by reducing apoptosis and increasing nitric oxide availability [67]. 

In addition, a great deal of evidence indicated that some miRNAs are directly involved in the control of the redox balance in endothelial cells. Along with mentioned above eNOS, NADPH oxidase (NOX), superoxide dismutase (SOD), glutathione peroxidase (GPx), and thioredoxin-dependent peroxidase (TrxR1) are essential enzymes for the maintenance of redox balance in cells. MiRNAs can regulate the function of NOX subunits. For example, hypercholesterolemia-induced miR-25 inhibition caused a significant increase in NOX4 expression levels in the heart leading to cardiac oxidative/nitrative stress and cardiac dysfunction [68]. Three miRNAs, such as miR-106b, miR-148b, and miR-204, by direct targeting of NOX2, showed a decrease in its expression levels in human and mouse cells [69]. NOX activation is the major source of reactive oxygen species in endothelial cells. Moreover, directly targeting SOD2 and SOD3, miR-21 displayed pro-oxidative effects [70,71]. Also, under oxidative stress conditions, downregulated miR-125a alleviated miR-125a-mediated translational repression of TrxR1, which, thereby, functioned as antioxidant defense [72]. The activity of GPx was increased by miR-133 overexpression that protects endothelial cells from oxidative stress-induced apoptosis [73]. In addition, miR-148a is likely to be involved in the oxidative stress-determined reduction of nitric oxide bioavailability. It can reduce eNOS activity via targeting rho-associated, coiled-coil containing protein kinase 1 (ROCK1), which contributes to early atherosclerotic lesion formation [74].

### 3.2. miRNAs in the Regulation of Endothelial Cell Senescence

Some miRNAs, including miR-134a, miR-217, miR-30, and miR-146a have important roles in the endothelial ageing. Vascular ageing is intimately involved in alterations in the biomechanical and structural properties of the vascular endothelial cells and vascular smooth muscle cells (VSMCs), thus, endothelial dysfunction, as well as increased arterial stiffness [75]. Particularly, endothelial cell senescence is important in atherosclerosis [76]. Endothelial cell alterations related to ageing associated with atherosclerosis include factors that promote atherogenesis, such as a decrease in compliance and an increase in the vascular inflammatory response. Thus, during ageing, progressively expressed in endothelial cells miR-134a and miR-217 promoted endothelial cell senescence via suppression of silent information regulator 1 (SIRT1), the key regulator of longevity and endothelial function [77,78]. On the other hand, miR-146a can delay endothelial cell senescence through the direct targeting of NADPH oxidase 4 (NOX4) protein that plays a protective role in the vasculature during ischemic or inflammatory stress [79]. Interestingly, targeting the same genes, different miRNAs can produce opposite effects, and this should be considered while analyzing the role of miRNAs. For example, mentioned above miR34a, by downregulating SIRT1, was able to promote endothelial cell senescence [77], whereas, let-7g produced the inhibitory effect on the senescence of endothelial cells through targeting the same SIRT1 gene [80].

### 3.3. miRNAs in Regulation of Endothelial Cell Apoptosis

Endothelial cell apoptosis plays a vital role in the initiation and progression of the development of the atherosclerotic lesion. The apoptosis of endothelial cells is responsible for plaque instability because it can predispose to arterial thrombosis that can lead to acute coronary occlusion and sudden death [81]. Accumulating evidence suggests that several miRNAs are implicated in regulatory mechanisms of endothelial cell apoptosis. Some have anti-apoptotic effects, while others are pro-apoptotic. The recent study demonstrated that apart from facilitating angiogenesis, the most endothelial cell abundant miR-126 can inhibit apoptosis of vascular endothelial cells through targeting important in regulating the cell cycle PI3K/Akt signaling pathway [82]. MiR-495 significantly promoted HUVEC proliferation by targeting chemokine (C-C motif) ligand 2 (CCL2) and inhibited apoptosis by affecting the cleaved caspase-3 expression [83]. On the contrary, by inhibiting SIRT1, miR-132 repressed proliferation, viability, and migration of tumor necrosis factor alfa (TNF-α)-induced HUVECs promoting apoptosis of these cells [84]. Moreover, endothelial apoptosis, likely contributing to the loss of endothelial cells, may expose surface extracellular matrix potentially stimulating platelet adherence and aggregation and the subsequent thrombus formation on the surface of the unstable plaque. In this respect, it was demonstrated that promoting endothelial cell apoptosis platelet-secreted miR-223, by targeting the insulin-like growth factor 1 (IGF-1) receptor, can participate in the formation of thrombus occurring in the later stage of atherosclerosis [85]. Noteworthy, some miRNAs can target several genes, therefore, one miRNA is able to regulate different cellular processes. For example, during the formation of atherosclerotic plaque, apart from the regulation of endothelial apoptosis, miR-223 can also participate in the development of the inflammatory response by regulating neutrophil function [86]. In addition, a new role of miR-30 mediating translational control of autophagy-related gene 6 (ATG6) in the regulation of endothelial cell autophagy during atherosclerosis was identified; it was shown that the elevated expression of the miR-30 can be caused by a high-fat diet that may suppress endothelial cell autophagy protective effects against atherosclerosis development [87].

### 3.4. miRNAs in the Regulation of Lipid Retention and Local Inflammation

MiRNAs are important regulators of lipid accumulation and development of local inflammation in the atherosclerotic plaque. The overexpression of miR-146a can delay both inflammatory response and oxidized low-density lipoprotein (oxLDL) accumulation by inhibiting the activation of toll-like receptor 4 (TLR4)-dependent signaling pathway [88]. MiR-125a-5p can also play a protective role in atherosclerosis by regulating the pro-inflammatory response, the uptake of lipids by macrophages (the process of transition of macrophages into foam cells), and the expression of oxysterol binding protein 9 (ORP9) in oxLDL-stimulated monocytes/macrophages [89]. Besides, this study showed that the inhibition of endogenous miR-125a-5p expression correlated with increased content of the following inflammatory cytokines: tumor growth factor-beta (TGF-β), tumor necrosis factor-alpha (TNF-α), interleukin 2 (IL-2), and interleukin 6 (IL-6) that determined the reduction of inflammation. Moreover, aberrant expression of miR-125a-5p can reduce expression of scavenger receptors of oxLDL-stimulated macrophages, such as the lectin-type oxidized LDL receptor 1 (LOX-1) and CD68, leading to a reduction of oxLDL-stimulated macrophage uptake and a consequent size reduction of atherosclerotic plaque [89]. The ORP9 activity mediates lipid metabolism and membrane transport producing the anti-atherogenic effect [90]. MiR-155 has a dual function in the regulation of the inflammatory process in the atherosclerotic plaque. The recent study indicated that increased expression of miR-155 can play an important role in the regulation of pro-inflammatory macrophage activity. It attenuated inflammation and the subsequent foam cell formation through miR-155/calcium-regulated heat stable protein 1 (CARHSP1)/TNF-α signaling pathway [91]. CARHSP1 participates in the oxidative stress response [92]. Moreover, miR-155 was shown to be involved in the post-transcriptional regulation of the inflammatory response via direct targeting of mitogen-activated protein kinase 10 (MAPK10) signaling pathway [93]. Likewise, miR-126 demonstrated the beneficial effect on inflammatory response also through MAP3K10 targeting [94]. On the other hand, miR-155 can contribute to the inflammatory processes promoting atherosclerotic disease [95]. It was suggested that pro-atherosclerotic effect of miR-155 can occur via targeting the suppressor of cytokine signaling 1 (SOCS1) in oxLDL-induced macrophages enhancing signal transducer and activator of transcription 3 (STAT3) and nuclear factor kappa-light-chain-enhancer of activated B cells (NF-κB) signaling [95]. Importantly, in this study, statistically significant inverse correlation between SOCS1 and miR-155 expression was observed indicating a significant biological function of SOCS1-miR-155 in atherosclerosis. STAT3 is a transcription factor that plays a major role in some cellular processes, such as cell growth and apoptosis [96]. In addition, other miRNAs, including, miR-31 and miR-17-3p can also regulate the development of vascular inflammation by controlling the expression of the adhesion molecules, such as vascular cell adhesion molecule 1 (VCAM-1), intercellular adhesion molecule (ICAM-1), and E-selectin [97].

### 3.5. miRNAs in the Regulation of VSMC Function

Rapid proliferation and growth of vascular cells occur after a non-specific vascular injury resulting in vascular hyperplasia and neointimal lesion formation. Neointimal lesion formation takes place at sites of subclinical atherosclerosis but it also is the established hallmark of restenosis after stenting, angioplasty, endarterectomy, arterial transplantation, and stroke [98,99]. Markedly, the increased proliferative ability of VSMCs is determined by the phenotypic switch of VSMCs from a contractile to a proliferative state that has arterial-wide implications to atherosclerotic plaques [100]. 

Several authors highlighted the influential role of miRNAs in regulating VSMC fate determination, plasticity, and neointimal lesion formation [101,102]. As a part of the larger proliferative response, the observations of miRNA profile alterations in balloon-injury and carotid-ligation models revealed a dynamic flux of some miRNAs in the arterial wall [102]. Thus, in neointimal formation models, miR-125a, miR-125b, miR-133, miR-143, miR-145, and miR-365 were observed to be downregulated, whereas, miR-21, miR-146, miR-214, and miR-352 were upregulated [102]. In fact, the role of miRNAs in VSMC proliferation was extensively studied [103,104,105,106]. Thus, miR-145 was demonstrated to be the main determinant of VSMC differentiation and phenotype switching, and to be downregulated in both atherosclerosis and arterial injury models [107,108]. These studies also proved the significant role of miR-143 in the regulation of VSMC function. Moreover, both miR-143 and miR-145, were shown to regulate the VSMC proliferative response to balloon-injury via alterations in cytoskeleton organization [109]. Another study showed that, in response to vascular damage, miR-143 and miR-145, as the components of a polycistronic cluster, can promote VSMCs contractile and inhibit proliferative (undifferentiated) phenotypes, therefore, suppress VSMCs proliferation and stabilize atherosclerotic plaque [110]. Depending on the muscle cell type, miR-133 emerged to have as positive as negative effects on the VSMC proliferation. The essential roles of miR-133 controlling VSMC phenotypic switch through the direct repression of the anti-proliferative and pro-apoptotic transcription factor Sp-1 in VSMCs were demonstrated in both in vitro and in vivo [111]. Similarly, the family of miR-133 was shown to play essential and redundant roles in the control of smooth (cardiac) muscle gene expression, hence, cardiomyocyte proliferation, differentiation, and cardiac growth [112]. Besides, the let-7 family were found to inhibit proliferation of VSMCs that can attenuate atherosclerotic process. Accordingly, reducing the expression of genes controlling cell proliferation, such as c-Myc and KRAS, let-7a decreased the VSMC proliferation in vitro and inhibited intimal hyperplasia in an experimental animal vein graft model [113]. Let-7d overexpression reduced VSMC growth by targeting cell signaling mediating gene KRAS [114]. Let-7g can significantly inhibit VSMC proliferation and migration induced by ox-LDL through targeting LOX-1 [115]. On the contrary, miR-221 and miR-222, apart from endothelial cells, were also abundantly expressed in VSMCs, in which they exhibited pro-atherosclerotic properties, i.e., verified in vivo effects of pro-proliferation and pro-migration [116]. The molecular mechanisms responsible for miR-221/222-mediated pro-atherosclerotic effects in VSMCs remain unclear.

### 3.6. MiRNAs in the Regulation of Calcification in VSMCs

Vascular calcification is a prominent feature of atherosclerosis and some miRNAs are implicated in the regulation of VSMC calcification. MiR-29a and miR-29b inhibited calcification of VSMCs by suppressing the expression of a disintegrin and metalloproteinase with thrombospondin motifs-7 (ADAMTS-7) [117]. Moreover, the study suggested that miR-125b downregulation can facilitate calcification of VSMCs by targeting Ets1, a transcription factor protein [118]. Besides, VSMC trans-differentiation into osteoblast-like cells can be promoted by the inhibition of endogenous miR-125b with the osteoblast transcription factor SP7 (osterix), as its target, which can regulate osteoblast differentiation [119]. Due to a limited amount of studies available on miRNA regulating the calcification process in atherosclerosis, it remains yet to be fully investigated.

### 3.7. miRNAs in the Regulation of Cholesterol Metabolism

Finally, miRNAs play a significant role in cholesterol metabolism and atherogenesis. Recently discovered co-transcribed with host genes miR-33a and miR-33b were described as key regulators of cholesterol and fatty acid homeostasis [120,121]. The studies showed that overexpression of miR-33 strongly inhibited expression of ATP-binding cassette transporter (ABCA1) at the RNA and protein level and reduced cellular cholesterol efflux to apolipoprotein A-I (ApoA-I), an important step in regulating reverse cholesterol transport [122,123]. ABCA1 contributes to the high-density lipoprotein (HDL) biogenesis in the liver, as well as its expression in macrophages is critical for reverse cholesterol transport [124]. Importantly, the inhibition of miR-33 expression in vivo resulted in a significant increase in expression of the hepatic ABCA1 and increased plasma HDL levels that facilitated atherosclerosis regression, therefore, confirmed the favorable physiological impact of miR-33 on the regulation of lipid metabolism [125]. Animal gene knockdown studies revealed that apart from the regulation of endothelial function, miR-122 also plays an important role in lipid metabolism [126,127]. Thus, in mice, inhibition of miR-122 expression by antisense oligonucleotides resulted in the increased oxidation of fatty acids in the liver and reduced cholesterol synthesis [126]. Besides, inhibition of miR-122 reduced total plasma cholesterol by about 35% encompassing beneficial alterations in both the LDL and HDL fractions [126]. Similarly, in African green monkeys treated with miR-122 antagomirs, wherein inhibition of miR122 caused a dose-dependent reduction in plasma cholesterol level without any signs of toxicity [127]. 

Several other miRNAs and their roles in atherosclerosis development were presented in Table 1.

The accumulated evidence indicated that miRNAs are tightly implicated in crucial pathomechanisms of atherosclerosis, including endothelial dysfunction, vascular angiogenesis, and remodeling, atherogenesis and lipid accumulation, local inflammation, thrombosis, and calcification. Possible mechanisms by which miRNAs correlate with atherosclerotic plaque formation and progression promise new hope in diagnosis, prognosis, and treatment of this disease.

**Figure 2:**
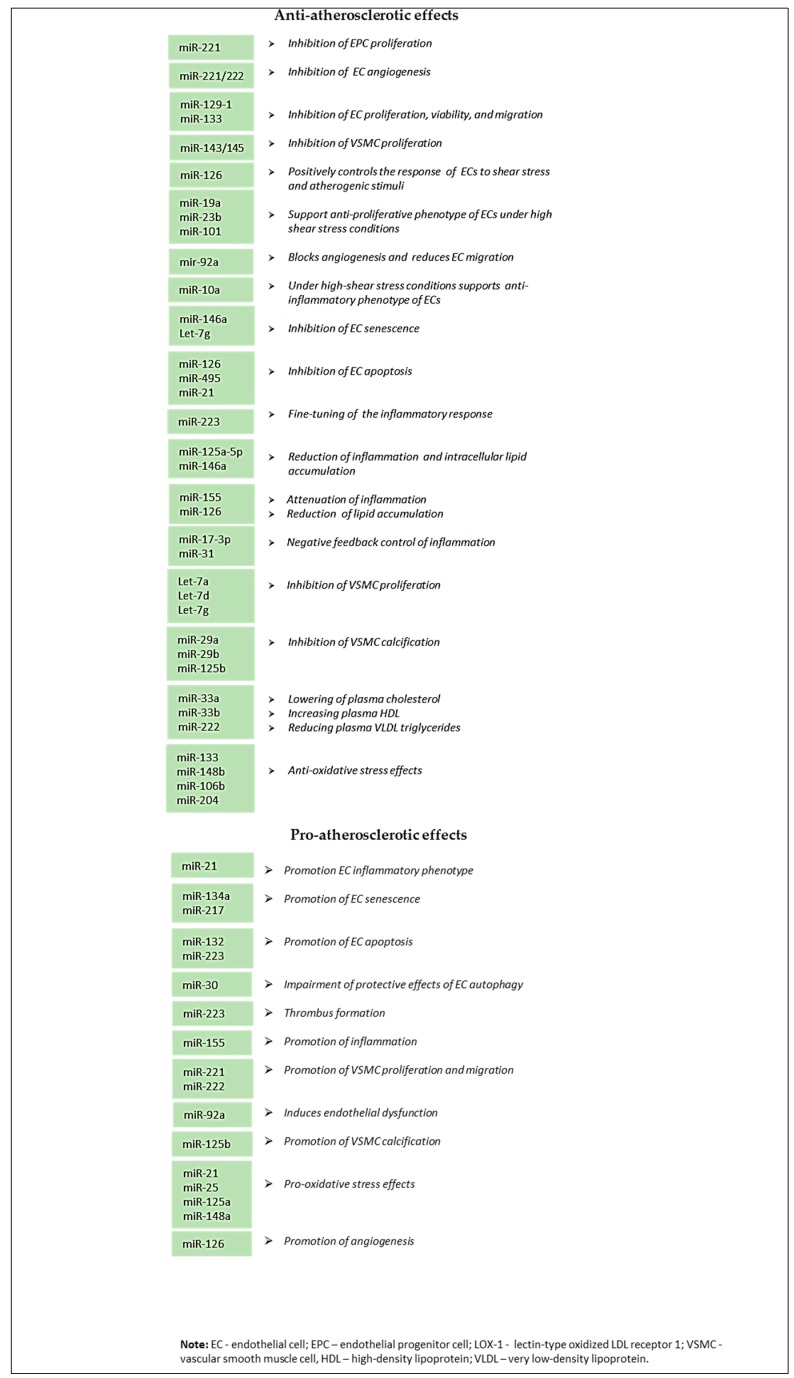
Summary of anti-atherosclerotic and pro-atherosclerotic effects of miRNAs.

The summary of anti-atherosclerotic and pro-atherosclerotic effects of described miRNAs was presented in Figure 2.

## 4. MiRNAs as Diagnostic Biomarkers in Atherosclerosis and CVDs

It was established that intracellular miRNAs may be released into body fluids, blood circulation including [150], although, the exact mechanisms of miRNA cellular release are still uncertain. Circulating miRNAs remain stable in serum and other body fluids because they are protected from degradation by RNases. Some researchers hypothesized that stability of circulating miRNAs may be determined by the high stability of the Ago2 protein and Ago2-miRNA complexes [151]; or due to loading into lipoprotein complexes, such as exosomes or micro-vesicles bound to HDL cholesterol particles [152,153]. In the past decade, a large amount of data was presented demonstrating that dysregulated levels of circulating miRNAs are strongly associated with the presence or absence of atherosclerosis or cardiovascular disease [154,155]. Though, the detection of mechanisms triggering the dysregulation and the putative effects of the shifts in circulating miRNAs levels continues to present challenges. Thus, circulating miRNA profiles showed that in serum samples of patients with obliterating atherosclerosis, there was the downregulation of miR-221, and miR-222 with the simultaneous upregulation of miR-27b, miR-130a, miR-21, and miR-210 [156]. These researchers also noted that dysregulated expression of these circulating miRNAs correlated with their aberrant expression levels in tissue samples obtained from the same atherosclerotic patients. Moreover, the levels of miR-126, miR-17, miR-92a, miR-145, and miR-155 were significantly lower in patients with CAD than in healthy controls [157]. The presence of dysregulated circulating levels of miR-1, miR-133a, miR-499, and miR-663 family associated with AMI was also reported [158,159]. Furthermore, it is believed that the evaluation of circulating expression levels of some miRNAs may serve as a valuable tool to assess the present vascular disease severity. The reduced expression of miR-210 leading to increased activity of the relevant target genes resulted in the substantial reduction of the fibrous cap stability of an atherosclerotic plaque [160]. In the view of that, this miRNA may have a clinical utility in the assessment of the risk of a carotid artery plaque rupture, particularly, in asymptomatic carotid atherosclerosis. Another human study showed that the expression levels of some miRNAs (miR-100, miR-127, miR-145, miR-133a, and miRNA-133b) in atherosclerotic plaques correlated with clinical signs of plaque destabilization supporting their prognostic significance for the risk stratification of vulnerable plaques [161]. Interestingly, miRNA expression profiling studies revealed that the expression of some miRNAs in the atherosclerosis-affected tissue can be significantly different in the serum of atherosclerotic patients [162]. It is important to note that expression levels of miRNAs may correlate with clinical indexes of atherosclerosis and that also may have a predictive/diagnostic value. The opposed relationship between miR-126 expression levels and circulating levels of LDL cholesterol was observed in patients with CAD [163]. Likewise, another clinical study confirmed that upregulated expression of miR-217 in plasma of subjects with atherosclerosis inversely correlated with serum levels of homocysteine and lipid parameters (triglycerides and LDL-cholesterol) [164].

Collectively, the findings indicated that specific expression patterns of miRNAs are linked to atherosclerosis or atherosclerosis-associated diseases, consequently, miRNAs have a strong potential to become novel diagnostic biomarkers. The major advantage of using miRNAs as biomarkers is the possibility for quantification of specific miRNAs by using quantitative reverse transcriptase-polymerase chain reaction (qRT-PCR), the standard technology for the detection and/or comparison of RNA levels with great specificity, sensitivity, and simplicity. Prospective large-scale human studies are required to validate the true potential of circulating miRNAs and changes in miRNA expression; hence, circulating miRNAs can serve as independent predictors of atherosclerotic diseases, and, moreover, whether other more readily accessible body fluids, such as urine or saliva, may be suitable for diagnosis.

## 5. RNA-Based Therapeutic Approaches for the Treatment of Atherosclerosis and CVDs

Provided the function of miRNAs in numerous pathways with pathophysiologic relevance to atherosclerosis, miRNAs emerged as fascinating candidates for the development of novel miRNA-based therapeutic strategies for this disease. Since they can target not only single mRNA but entire networks of frequently functionally-associated genes, it is possible to make a considerable impact on the signal-dependent tissue remodeling and spare negative effects on tissue function. In this way, the following classes of RNA-based therapeutics were proposed to be potentially effective to counteract disturbed gene expression and impaired vascular function: antisense oligonucleotide (anti-miRs) and miRNA mimetics and inhibitors. Antisense oligonucleotides possess a miRNA silencing ability that modifies specific pathways or reduces dysregulated expression. It was demonstrated by preclinical studies that targeting both miR-33a and miR-33b by systemic delivery of an anti-miR oligonucleotide increased hepatic expression of ABCA1 resulting in a persistent increase in plasma HDL and lowering of VLDL triglycerides in African green monkeys [165]. Similar results were presented by another study that also used non-human primates [127]. It was shown that the simple systemic delivery of oligonucleotide, such as locked nucleic acid (LNA)-anti-miR, effectively antagonized miR-122 expression in the liver leading to a prolong and reversible decrease in total plasma cholesterol without any recorded LNA-related toxicities or histopathological modifications in tissues of the study animals. Relying on data obtained with the use of these animal models of atherosclerotic disease, the promising therapeutic method utilizing anti-miRs for the treatment of dyslipidemias can be developed. Using the delivery of miRNA mimics or inhibitors is another attractive therapeutic approach in the aid of reconstituting of a miRNA expression dysregulated by a disease. For example, in the aortic intima and plasma of apolipoprotein E–deficient mice, miR-181b was markedly downregulated, and systemic delivery of miR-181b mimics produced the 2.3-fold increase in the miR-181b expression that significantly repressed the formation of the atherosclerotic lesion [166]. Correspondingly, a downregulated miR-181b expression is attributed to patients with CAD [167]. Another possible method for inhibition of miRNAs involves the use of miRNA sponges or decoy transcripts containing multiple binding sites complementary to a miRNA of interest. The presented evidence indicated that binding of miRNA sponges to target miRNA can inhibit its function, and this method can be as effective as the antisense technology [168]. This method can be also used for validation of target predictions and assay miRNA loss-of-function phenotypes [168]. Additionally, further possible therapeutic approach of either antagonizing or mimicking miRNA actions may include cell-based therapy [169,170]. The employment of specific anti-miRs, miRNA mimics, and target site blockers enabled performing a large number of in vitro and in vivo experiments, elucidating the role of miRNAs and their potential application as diagnostic and/or prognostic biomarkers, as well as drug targets. Despite of most of the data on the role of miRNAs in cardiovascular pathophysiology is still preliminary, the significant progress was achieved in the development of RNA therapeutics for the treatment of atherosclerosis. Mipomersen, an antisense oligonucleotide drug designed to treat homozygous familial hypercholesterolemia, showed beneficial results in clinical trials [171]. Later it was approved by the Food and Drug Administration agency in the United States [172]. Moreover, in the United States, 4% of all commercial patents disclosing the RNA-based medicinal preparation filings belong to CVDs [173].

However, the continuing problem of any miRNA-based therapy is to achieve effective and steady targeted delivery of pharmacological compounds to miRNA targets. In this respect, the stability, affinity to target cells, and uptake by tissues of anti-miRs can be chemically enhanced. The use of LNA, a new class of chemically engineered oligonucleotides, showed the strong binding to a miRNA [174]. Remarkably, attempting to increase the cellular uptake, analogues of cholesterol were added to anti-miRs, and that promoted their integration into LDL and HDL [175]. The detailed evaluation of chemical modifications will be required to diminish potential adverse effects and unexpected toxicities. Using the high-affinity to the heart adeno-associated virus (AAV) vectors, such as AAV9, can enhance specificity of miRNA targeting without adverse effects [176]. There are other pending issues related to successful target delivery. Many target genes of specific miRNAs are not validated, and their mechanisms of action are uncertain. Also, in silico prediction of many target genes may be irrelevant in vivo due to lower physiological concentrations, or inconsistencies in localization between miRNAs and their potential targets. The analysis of the miRNA quantification by qRT-PCR is lacking optimization of analytical methods and that may produce false positives results. Therefore, a large amount of further work is required to establish whether therapeutic manipulation of miRNA function may indeed represent a safe and efficient cure of atherosclerosis that can be adopted by clinics.

## 6. Conclusions

The research of critical roles of miRNAs in atherosclerosis development is an emerging field, nevertheless, the numbers of miRNAs targeting the various aspects of atherosclerosis pathogenesis are rapidly growing. To date, accumulated evidence indicated that many miRNAs can make an indisputable impact on atherosclerosis initiation, progression, and the development of its complications. Therefore, they were considered as potential clinical biomarkers and had already begun to be implemented as promising novel therapeutic targets with the intent of providing patients with better management of atherosclerosis and CVDs.

## Figures and Tables

**Table 1:** miRNAs involved in the atherosclerosis pathogenesis.

miRNA	Target	Function	References
miR-27a/b	SEM6A	Regulates sprouting angiogenesis of endothelial cells	[128]
miR-126-5p	SetD5 Dlk1 ALCAM	Promotes endothelial cell proliferation and repair; regulates leucocyte adhesion and transmigration	[129,130]
miR-126	G protein	Reduces the macrophage and apoptotic cell content of plaques leading to lesion reduction in size with a less inflammatory phenotype; governs vascular integrity and angiogenesis	[131]
miR-302a	ABCA1	Regulates cellular cholesterol efflux in macrophages	[132]
miR-758	ABCA1	Regulates macrophage cholesterol efflux protecting cells from the excessive lipid accumulation	[133]
miR-342-5p	AKT1	Enhances proinflammatory macrophage mediators, such as iNOS and IL-6, via suppression of miR-155 expression	[134]
miR-370		Regulates lipid metabolism by mediating synthesis of cholesterol, fatty acid, and fatty acid β-oxidation; regulates miR-122 levels	[135]
miR-10a	MAP3K7, β-TRC	Inhibits NF-κB activation	[136]
miR-663	JunB Myosin light chain 9	Regulates monocyte adhesion induced by oscillatory shear stress, therefore, it involved in oscillatory shear stress-induced cellular inflammation; regulates VSMC phenotypic switch and vascular neointimal formation	[137,138]
miR-210	EFNA3 PDK1	Linked to intraplaque angiogenesis and, possibly, to the formation of unstable plaques; regulates endothelial apoptosis	[139,140]
miR-34a	PNUTS	Regulates cardiac aging and contractile function	[141]
miR-424/322	Calumenin STIM1 Cyclin D1	Regulates proliferation, migration, and differentiation of VSMCs	[142]
miR-23b	E2F1 Rb	Inhibits endothelial cell proliferation	[143]
miR-29	ADAMTS-7	Mediates VSMC calcification	[117]
miR-147	TLR2 TLR3 TLR4	Negatively regulates the TLR-associated signaling pathway increasing inflammatory cytokine expression in macrophages, hence, prevents excessive inflammatory responses	[144]
miR-181a	c-Fos OPN	Diminishes the oxLDL-induced immune-inflammatory response by downgrading dendritic maturation of cell surface molecules, such as CD40 and CD83; regulates endothelial cell proliferation; protects against angiotensin II-induced osteopontin expression in VSMCs	[145,146]
miR-181b	NF-κB	Suppresses the endothelial inflammatory response	[147]
miR-9	ACAT1	Regulates cellular cholesterol homeostasis by decreasing the formation of foam cells from macrophages	[148]
miR-200c	ZEB1	Upregulated upon oxidative stress, induces apoptosis and senescence of endothelial cells	[149]

Note: ABCA1—ATP-binding cassette transporter 1; ACAT1—acetyl-CoA acetyltransferase 1; ADAMTS-7—a disintegrin and metalloproteinase with thrombospondin motifs 7; Akt1 – RAC - alpha serine/threonine-protein kinase encoded by the AKT1 gene in humans; ALCAM—CD166 antigen encoded by the ALCAM gene in humans; β-TRC—β-transducin repeat-containing gene; c-Fos – transcription factor; Calumenin—calcium-binding protein; Cyclin D1—protein involved in regulation of cell cycle progression; Dlk1—delta-like 1 homolog; E2F1—transcription factor controlling cell cycle; EFNA3—Ephrin A3 protein; G protein—heterotrimeric guanosine triphosphate–binding protein; ICAM 1—intercellular adhesion molecule 1; iNOS—inducible nitric oxide synthase; JunB—transcription factor; MAP3K7—mitogen-activated protein kinase 7; OPN—osteopontin; oxLDL—oxidized low-density lipoprotein; PDK1—pyruvate dehydrogenase kinase isoform; PNUTS—serine/threonine-protein phosphatase 1 regulatory subunit 10 encoded by the PNUTS gene; Rb—retinoblastoma protein controlling cell cycle; RGS16—regulator of G-protein signaling; SEM6A—semaphorin-6A; SetD5—SET domain containing 5 protein; SIRT1—NAD-dependent deacetylase sirtuin-1 encoded by the SIRT1 gene in humans; STIM1—stromal interaction molecule 1; TLR2,3,4—toll-like receptor 2,3,4; TNF-α—tumor necrosis factor alpha; VSMCs—vascular smooth muscle cells; ZEB1—zinc finger E-box-binding homeobox 1 protein.
